# Association between Hardness (Difficulty of Chewing) of the Habitual Diet and Premenstrual Symptoms in Young Japanese Women

**DOI:** 10.4137/EHI.S2810

**Published:** 2010-01-18

**Authors:** Kentaro Murakami, Satoshi Sasaki, Yoshiko Takahashi, Kazuhiro Uenishi, Tomoko Watanabe, Toshiyuki Kohri, Mitsuyo Yamasaki, Reiko Watanabe, Keiko Baba, Katsumi Shibata, Toru Takahashi, Hitomi Hayabuchi, Kazuko Ohki, Junko Suzuki

**Affiliations:** 1 Department of Social and Preventive Epidemiology, School of Public Health, the University of Tokyo, Tokyo 113-0033, Japan; 2 Department of Health and Nutrition, School of Home Economics, Wayo Women’s University, Chiba 272-8533, Japan; 3 Laboratory of Physiological Nutrition, Kagawa Nutrition University, Saitama 350-0288, Japan; 4 Department of Nutrition, Chiba Prefectural University of Health Science, Chiba 261-0014, Japan; 5 Department of Food Science and Nutrition, School of Agriculture, Kinki University, Nara 631-8505, Japan; 6 Department of Health and Nutrition Science, Faculty of Health and Social Welfare Science, Nishikyushu University, Saga 842-8585, Japan; 7 Department of Health and Nutrition, Faculty of Human Life Studies, University of Niigata Prefecture, Niigata 950-8680, Japan; 8 Department of Nutrition, Mie Chukyo University Junior College, Mie 515-8511, Japan; 9 Laboratories of Food Science and Nutrition, Department of Life Style Studies, School of Human Cultures, The University of Shiga Prefecture, Shiga 522-8533, Japan; 10 Graduate School of Human Life Science, Mimasaka University, Okayama 708-8511, Japan; 11 Department of Human Environmental Science, Fukuoka Women’s University, Fukuoka 813-8529, Japan; 12 Graduate School of Science for Living System, Showa Women’s University, Tokyo 154-8533, Japan; 13 Department of Health and Nutrition, Faculty of Human Sciences, Hokkaido Bunkyo University, Hokkaido 061-1449, Japan. Email: stssasak@m.u-tokyo.ac.jp

**Keywords:** hardness of diet, chewing, premenstrual symptoms, epidemiology

## Abstract

Recent evidence suggests that voluntary rhythmic movements such as chewing may increase blood serotonin and subsequently brain serotonin, which in turn acts to alleviate premenstrual symptoms. In this observational cross-sectional study, we tested the hypothesis that hardness (difficulty of chewing) of the habitual diet (i.e. dietary hardness) is associated with decreased premenstrual symptoms. Subjects were 640 female Japanese dietetic students aged 18–22 years. Dietary hardness was assessed as an estimate of masticatory muscle activity for the habitual diet (i.e. the difficulty of chewing the food). The consumption of a total of 107 foods was estimated by means of a self-administered, comprehensive diet history questionnaire, and masticatory muscle activity during the ingestion of these foods was estimated according to published equations. Menstrual cycle symptoms were assessed using the retrospective version of the Moos Menstrual Distress Questionnaire, from which total score and subscale scores (i.e. pain, concentration, behavioral change, autonomic reactions, water retention, and negative affect) in the premenstrual phase were calculated and expressed as percentages relative to those in the intermenstrual phase. Dietary hardness was not associated with total score in the premenstrual phase (*P* for trend = 0.48). Further, no association was seen for any subscale score in the premenstrual phase (*P* for trend = 0.18–0.91). In conclusion, this preliminary study failed to substantiate a hypothesized inverse relationship between hardness of the habitual diet and premenstrual symptoms. Considering the plausibility of the putative mechanism, however, further investigation using more relevant measures of chewing and premenstrual symptoms is warranted.

## Introduction

Premenstrual symptoms are characterized by a set of behavioral, somatic, and affective symptoms of varying severity which occur during the 7–10 days prior to the onset of menstruation and subside after the beginning of the menstrual flow. Although the etiology of premenstrual symptoms is largely unknown, current evidence suggests that serotonin may be important in the pathogenesis of premenstrual symptoms; reduction in brain serotonin neurotransmission is thought to lead to mood and behavioral symptoms associated with premenstrual symptoms, such as poor impulse control, depressed mood, and irritability.^1^–^3^ Another line of evidence suggests that voluntary rhythmic movements such as chewing may increase blood serotonin, and subsequently brain serotonin; Mohri and colleagues found that blood (platelet poor plasma and whole blood) serotonin levels increased in response to chewing.^4^ On this basis, chewing might be expected to alleviate premenstrual symptoms by increasing brain serotonin, although it is unknown whether chewing would affect all premenstrual symptoms similarly or affect specifically some symptoms. To our knowledge, however, the relation between chewing and premenstrual symptoms has not been investigated. In this observational cross-sectional study, we investigated the associations between hardness (difficulty of chewing) of the habitual diet (i.e. dietary hardness; an approximate measure of habitual chewing) and premenstrual symptoms. We hypothesized that dietary hardness is associated with decreased premenstrual symptoms.

## Methods

### Subjects

The present study was based on a cross-sectional multi-center survey conducted from January to March 2007 among female dietetic students from 11 institutions in Japan. All measurements at each institution were conducted according to the survey protocol. Staff at each institution explained an outline of the survey to potential subjects. Those who agreed to participate were then provided detailed written and oral explanations of the survey’s general purpose and procedure. The protocol of the study was approved by the Ethics Committee of the National Institute of Health and Nutrition, and written informed consent was obtained from each subject, and also from a parent for subjects aged < 20 years.

A total of 702 Japanese women took part. For the present analysis, women aged 18–22 years were selected (*n* = 687). We then excluded women not completing the survey questionnaires (*n* = 1), those not completing anthropometric measurements (*n* = 2), those who had been pregnant at any time in the preceding year (*n* = 3), those with diagnosed endometabolic diseases such as diabetes and thyroid diseases (*n* = 4), those currently taking oral contraceptives (*n* = 7) or steroid hormones (*n* = 16), those who had few or no menstruations during the preceding year (*n* = 9), those currently receiving dietary counseling from a doctor or dietitian (*n* = 7), and those with extremely low or high reported energy intakes (<500 or >4000 kcal/day; *n* = 2). As some women fell into more than one exclusion category, the final sample comprised 640 women.^5^

### Dietary hardness

Dietary habits during the preceding month were assessed using a self-administered, semi-quantitative, comprehensive diet history questionnaire (DHQ).^6^–^9^ Responses to the DHQ, as well as those to an accompanying lifestyle questionnaire, were checked at least twice for completeness by trained survey staff (mostly registered dietitians) and, when necessary forms were reviewed, with the subject to ensure the clarity of answers.

The DHQ is a structured 16-page questionnaire that consists of the following seven sections: general dietary behavior; usual cooking methods; frequency of consumption and amount of selected alcoholic beverages; frequency of consumption and semi-quantitative portion size of selected food and non-alcoholic beverage items; dietary supplements; frequency of consumption and semi-quantitative portion size of selected staple foods (e.g. rice, bread, and noodles), soup consumed with noodles, and miso (i.e. fermented soybean paste) soup; and open-ended items for foods consumed regularly (at least once/week) but not appearing in the DHQ.^6^ The food and beverage items were selected as foods commonly consumed in Japan, mainly from a food list used in the National Nutrition Survey of Japan, and standard portion sizes were derived mainly from several recipe books for Japanese dishes.^6^ Estimates of daily intake for foods and beverages (150 items in total) and energy were calculated using an ad hoc computer algorithm for the DHQ,^6^,^7^ based on the Standard Tables of Food Composition in Japan.^10^ Information on dietary supplements and data from the open-ended questionnaire items were not used in the calculation of dietary intake.^6^

Satisfactory validity of the DHQ with respect to commonly studied nutritional factors (e.g. energy-adjusted nutrient intake) has been shown in several previous studies.^6^–^9^ Briefly, Pearson correlation coefficients were 0.48 for energy, 0.37–0.75 for energy-providing nutrients, and 0.38–0.68 for other nutrients between the DHQ and 3-day estimated dietary records in 47 women;^6^ 0.23 for sodium and 0.40 for potassium between the DHQ and 24-hour urinary excretion in 69 women;^8^ 0.66 between the DHQ and serum phospholipid concentrations for marine-origin n-3 polyunsaturated fatty acids (sum of eicosapentenoic, docosapentaenoic, and docosahexaenoic acids) in 44 women;^9^ and 0.56 between the DHQ and serum concentrations for carotene in 42 women.^9^

In the present study, dietary hardness was assessed as estimated masticatory muscle activity needed for the habitual diet (i.e. the difficulty of chewing the food in the diet).^11^ Whereas the habitual diet was assessed by DHQ^6^–^9^ as described above, estimates of masticatory muscle activity for each food in the DHQ were obtained from equations published by Yanagisawa and colleagues.^12^ Those authors measured the activities of 6 muscle regions (mV·s) involved in mastication (right and left masseters and anterior and posterior temporalis) by using electromyography during the ingestion of the same volume (1.3 × 1.3 × 1.3 cm) of 16 selected foods with various physical properties by 20 healthy Japanese adults (10 men and 10 women) with a mean age of 21 years. They found that masticatory muscle activities (mV·s/2.197 cm^3^) were highly correlated with the physical properties of foods (i.e. firmness, cohesiveness, and strain) as measured with a texturometer (GTX-2; ZenkenKKInc, Chiba, Japan) and developed the following equations:^12^

$$\begin{array}{l} \text{Masticatory}\;\text{muscle}\;\text{activity}=0.6586\times \text{ln}\;(\text{firmness}\\ \times \text{cohesiveness}\;\times \text{strain}\times 10)-0.0307\\ \text{where}\;R^{2}=0.89;\\ \text{Masticatory}\;\text{muscle}\;\text{activity}=0.2718\times \text{firmness}\\ +0.0335\;\times \text{strain}-0.0030\\ \text{where}\;R^{2}=0.89;\;\text{or}\\ \text{Masticatory}\;\text{muscle}\;\text{activity}=0.3081\times \text{firmness}\\ +0.3300\\ \text{where}\;R^{2}=0.81. \end{array}$$

Using the information on the physical properties of foods they had measured earlier with a texturometer,^13^ Yanagisawa and colleagues^12^ then estimated masticatory muscle activities for a total of 144 foods according to one of their equations, by using the available variables (i.e. firmness, cohesiveness, and strain).

They did not, however, cross-validate the equations to show their applicability.^12^ We therefore conducted a cross-evaluation^11^ by using data reported by Shiono and colleagues.^14^ Those authors measured the activities of 4 muscle regions (mV·s) involved in mastication (right and left masseters and anterior temporalis, but not posterior temporalis) by using electromyography during the ingestion of standard-sized bites (2.4–44.5 g) of 46 selected foods with various physical properties by 6 healthy Japanese adults (3 men and 3 women) aged 23–27 years. By careful direct matching, information on masticatory muscle activities for a total of 18 foods was available from Shiono and colleagues^14^ and information on physical properties was available from Yanagisawa and colleacues.^13^ Pearson correlation coefficient between masticatory muscle activities measured by Shiono and colleagues (mV·s/g food)^14^ and those estimated by using physical property values as described by Yanagisawa and colleagues (mV·s/g food (= mV·s/2.197 cm^3^ divided by 2.197, assuming that the density of all foods = 1))^12^ was 0.88 among these 18 foods.^11^ This high correlation suggests the applicability of the equations developed by Yanagisawa and colleagues,^12^ despite the differences in masticatory muscles measured and in the amounts of foods consumed in the studies of Yanagisawa and colleagues^12^ and Shiono and colleagues.^14^

During the calculation of dietary hardness, we included solid foods only (107 items on the DHQ), and excluded liquid foods, including beverages, and seasonings, including fats and oils (43 items).^11^ We directly matched each of 107 solid food item on the DHQ^6^–^9^ with foods for which information on masticatory muscle activities was available (*n* = 144 items) from Yanagisawa and colleagues.^12^ Foods for which masticatory muscle activities had not been determined (21 items) were assigned a value according to that of a comparable food.^11^

Because the physical properties (and hence the hardness, or difficulty of chewing) of vegetables are greatly influenced by cooking with heat,^12^ we took those influences into account as much as possible.^11^ For tomatoes and cucumbers, we used values for raw tomatoes and raw cucumbers, respectively, because these vegetables are usually consumed without heating in Japan. For cabbage, we used a weighted mean of a value for raw cabbage and that for boiled leafy vegetables (because of a lack of information on boiled cabbage), based on the ratio of the observed consumption (g/day) of raw cabbage to that of cabbage cooked with heat (i.e. 4:6) in 16-day weighed dietary records conducted by 92 women (S. Sasaki, unpublished observations, 2006). For carrots, we used a weighted mean of a value for raw carrots and that for boiled carrots, based on the ratio of the observed consumption (g/day) of raw carrots to 5 that of carrots cooked with heat (i.e. 3:7) in 92 women (S. Sasaki, unpublished observations, 2006). For other vegetables, we used values adjusted for cooking with heat, given that these foods are usually consumed after cooking with heat in Japan.

Dietary hardness was calculated as the sum of the products of estimated masticatory activities (mV·s/2.197 cm^3^) and the volume of food consumed (cm^3^/day) divided by 2.197.^11^ For the estimation of food volume, we simply converted weight in grams to weight in cubic centimeters for all of the foods, on the assumption that the density of all foods = 1. Because the crude value of dietary hardness was strongly correlated with energy intake (Pearson correlation coefficient: 0.75), the energy-adjusted value (mV·s/4184 kJ) was used in the present study.^11^ Estimates of masticatory muscle activity for the 107 food items used to calculate dietary hardness have been presented previously.^11^

We could not investigate the relative validity of the DHQ in assessing dietary hardness against 16-day dietary records, because an insufficient number of foods (*n* = 144 items) with information on hardness (i.e. masticatory muscle activity)^12^ prevented the calculation of dietary hardness by 16-day dietary records.^11^

### Premenstrual symptoms

Menstrual cycle symptoms during the preceding year were assessed using the Japanese version^15^ of Magos and colleagues’ modification^16^ of the retrospective version of the Moos Menstrual Distress Questionnaire (MDQ).^17^ The MDQ, incorporated into the lifestyle questionnaire, consists of a total of 45 symptom items,^16^ which are grouped into eight subscales:^17^ pain, concentration, behavioral change, autonomic reactions, water retention, negative affect, arousal, and non-specific adverse symptoms designed to detect those experiencing symptoms (control). Each symptom item was rated by each subject on a 5-point scale from 1 (no experience of the symptom) to 5 (disabling or incapacitating experience of the symptom),^15^ separately for the three menstrual cycle phases [menstrual (during menstrual flow), premenstrual (the week before the beginning of menstrual flow), and intermenstrual (remainder of cycle) phases].^17^ The MDQ scores were calculated for each subscale and the total score (excluding arousal and control) for each cycle phase.^15^,^18^ The total and subscale MDQ scores in the premenstrual phase expressed as percentages relative to those in the intermenstrual phase were used in the present study.^5^

### Other variables

Body height was measured to the nearest 0.1 cm with the subject standing without shoes. Body weight in light indoor clothes was measured to the nearest 0.1 kg. Body mass index was calculated as body weight (kg) divided by the square of body height (m). In the lifestyle questionnaire, the subject reported her residential area, which was grouped into one of three regions [residential block: north (Kanto, Hokkaido, and Tohoku), central (Kinki, Tokai, and Hokuriku), or south (Kyushu and Chugoku)]. The residential areas were also grouped into three categories according to population size (size of residential area: city with population ≥1 million, city with population <1 million, or town and village). Current smoking (yes or no), age at menarche, whether currently experiencing menstrual flow, date of the start of the last (or current) menstrual flow, usual length of the menstrual cycle, and usual number of days of bleeding were also self-reported in the lifestyle questionnaire. For women who reported irregular menstrual cycles, we asked the range of the length of cycles and allotted the median as the cycle length.^19^ According to information on whether the subject was currently menstruating, the date of the start of the last menstruation, and the usual length of the menstrual cycle, as well as the date the lifestyle questionnaire was completed, the subjects were divided into three categories of menstrual cycle phase as at the time of the study (menstrual, premenstrual, or intermenstrual phases). Physical activity was calculated as the average metabolic equivalent-hours per day^20^ on the basis of the frequency and duration of five different activities (sleeping, high- and moderate-intensity activities, walking, and sedentary activities) over the preceding month, as reported in the lifestyle questionnaire.^21^ Dietary glycemic index (a measure of carbohydrate quality) was calculated by multiplying the contribution of each individual food to the daily intake of available carbohydrate by the food’s glycemic index value and then summing the product, based on a total of 72 major carbohydrate-containing foods.^5^

### Statistical analysis

All statistical analyses were performed using SAS statistical software version 9.1 (SAS Institute Inc, Cary, NC, USA). Data were presented as mean and SD for continuous variables including dietary hardness and as percentage for categorical variables. Dietary hardness was also described as median and range according to quintile of dietary hardness. Using the PROC GLM procedure, linear regression models were constructed to examine the association of dietary hardness (in quintiles) with premenstrual symptom scores, and mean (and SE) total and subscale MDQ scores in the premenstrual phase were calculated by quintiles of dietary hardness after multivariate adjustment for potential confounding factors, including age, body mass index, residential block, size of residential area, current smoking, age at menarche, usual length of the menstrual cycle, usual number of days of bleeding, menstrual cycle phase at the time of the study, physical activity, and dietary glycemic index.^5^ Linear trends with increasing levels of dietary hardness were tested by assigning each participant a median value for the category and modeling this value as a continuous variable. All reported *P* values are two-tailed, and *aP* value of <0.05 was considered statistically significant.

## Results

Basic characteristics of subjects are shown in Table 1. Mean dietary hardness was 175 (SD: 31) mV·s/4184 kJ. Mean total MDQ score in the premenstrual phase relative to that in the intermenstrual phase was 125.6% (SD: 34.2%). Selected characteristics according to quintile of dietary hardness are shown in Table 2. There was a positive association between dietary hardness and usual number of days of bleeding. Dietary hardness was positively associated with dietary glycemic index.

MDQ scores in the premenstrual phase according to quintile of dietary hardness are shown in Table 3. Because results for the crude and multivariate analyses were similar for all variables analyzed, we present hear only those derived from the multivariate models. Dietary hardness was not associated with total MDQ score in the premenstrual phase. Further, no association was seen for any of the subscale MDQ scores in the premenstrual phase, including pain, concentration, behavioral change, autonomic reactions, water retention, and negative affect.

## Discussion

Although the etiology of premenstrual symptoms is largely unknown, current evidence suggests that they might arise from a decrease in brain serotonin neurotransmission.^1^–^3^ Another line of evidence suggests that chewing may increase blood serotonin and subsequently brain serotonin.^4^ On this basis, chewing might be expected to alleviate premenstrual symptoms by increasing brain serotonin. Despite the putative mechanism, however, we saw no evident association between hardness (difficulty of chewing) of the habitual diet (i.e. dietary hardness; an approximate measure of habitual chewing) and premenstrual symptoms in this study of young Japanese women. To our knowledge, this study is the first to examine the relationship of measures of chewing with premenstrual symptoms. The null findings may be at least partly explained by several limitations of the study, including its novel, as yet unvalidated method of assessing dietary hardness, reliance on self-reports for assessing premenstrual symptoms, and cross-sectional design. Alternatively, given that premenstrual symptoms are associated with a wide range of physiological and behavioral factors, the influence of dietary hardness on symptoms may not necessarily be detectable.

Several limitations of the study should be acknowledged. First, its cross-sectional nature did not permit the assessment of causality owing to the uncertain temporality of the association. Second, our subjects were selected female dietetic students, not a random sample of Japanese women, and thus our results might not apply to the general Japanese population. Third, the validity of estimated dietary hardness is unknown as the method used is novel and yet to be established, and the procedure we used provides only an approximation of the actual hardness of habitual diet.^11^ Additionally, we used estimated dietary hardness as an approximate measure of habitual chewing; investigation based on more relevant measures of chewing is undoubtedly needed. Fourth, premenstrual symptoms were assessed using a retrospective questionnaire,^17^ which may provide an inflated estimation of symptom severity and be heavily reliant on memory of past menstrual-related symptoms. Fifth, dietary hardness and menstrual symptoms were evaluated in different periods, namely in the preceding month for the former and in the preceding year for the latter, albeit that the results did not materially change when analysis was limited to women reporting a stable diet with the preceding year (*n* = 526; data not shown). Sixth, although adjustments were made for a variety of potential confounding factors, residual confounding could not be ruled out; in particular, no consideration was given to stress.^5^,^22^ Finally, the calculation of the statistical power, which is needed for presenting null findings, was impossible because of the lack of information on the effect size.

In conclusion, this preliminary study failed to substantiate the hypothesized inverse relationship between hardness of the habitual diet and premenstrual symptoms. Considering the plausibility of the putative mechanism, however, further investigation using more relevant measures of chewing and premenstrual symptoms is warranted.

## Figures and Tables

**Table 1:** Basic characteristics of 640 Japanese women aged 18–22 years.

**Variable**	**Value**
Age (years)	19.7 ± 1.1
Body height (cm)	158.5 ± 5.4
Body weight (kg)	53.7 ± 7.4
Body mass index (kg/m^2^)	21.4 ± 2.5
Age at menarche (years)	12.3 ± 1.4
Usual length of menstrual cycle (days)	31.8 ± 11.1
Usual number of days of bleeding	6.1 ± 1.2
Physical activity (total metabolic quivalents-hours/day)	33.8 ± 2.8
Dietary glycemic index	65.4 ± 4.0
Dietary hardness (mV·s/4184 kJ)	175 ± 31
MDQ total score in premenstrual phase (%)^b^	125.7 ± 34.2
MDQ subscale scores in premenstrual phase (%)^b^	
Pain	132.7 ± 41.6
Concentration	114.1 ± 33.7
Behavioral change	129.4 ± 52.1
Autonomic reactions	106.9 ± 25.8
Water retention	143.6 ± 58.5
Negative affect	130.4 ± 54.6

a Values are means ± SDs.

b MDQ scores in premenstrual phase were expressed as percentages relative to those in the intermenstrual phase.

**Abbreviations:** MDQ, Menstrual Distress Questionnaire.

**Table 2:** Selected characteristics according to quintile of dietary hardness in 640 Japanese women aged 18–22 years.^a^

**Variable**	**Quintile 1 *(n* = 128)**	**Quintile 2 *(n* = 128)**	**Quintile 3 *(n* = 128)**	**Quintile 4 *(n* = 128)**	**Quintile 5 *(n* = 128)**	***P*^b^**
Dietary hardness (mV·s/4184 kJ)	133 ± 16	159 ± 6	177 ± 4	192 ± 4	217 ± 19	—
Age (years)	19.6 ± 1.0	19.7 ± 1.1	19.8 ± 1.1	19.6 ± 1.1	19.7 ± 1.1	0.22
Body mass index (kg/m^2^)	21.5 ± 2.5	21.5 ± 2.4	21.5 ± 2.6	21.3 ± 2.5	21.0 ± 2.7	0.09
Residential block						0.63
North (Kanto, Hakkaido, and Tohoku)	72 (56.3)	71 (55.5)	66 (51.6)	75 (58.6)	65 (50.8)	
Central (Kinki, Tokai, and Hokuriku)	36 (28.1)	34 (26.6)	37 (28.9)	31 (24.2)	41 (32.0)	
South (Kyushu and Chugoku)	20 (15.6)	23 (18.0)	25 (19.5)	22 (17.2)	22 (17.2)	
Size of residential area						0.33
City with population >1 million	15 (11.7)	17 (13.3)	16 (12.5)	25 (19.5)	25 (19.5)	
City with population <1 million	109 (85.2)	107 (83.6)	110 (85.9)	92 (71.9)	96 (75.0)	
Town and village	4 (3.1)	4 (3.1)	2 (1.6)	11 (8.6)	7 (5.5)	
Current smokers	2 (1.6)	3 (2.3)	6 (4.7)	2 (1.6)	1 (0.8)	0.57
Age at menarche (years)	12.4 ± 1.5	12.4 ± 1.5	12.2 ± 1.3	12.2 ± 1.2	12.4 ± 1.6	0.53
Usual length of menstrual cycle (days)	33.4 ± 15.2	31.3 ± 10.5	30.3 ± 8.5	31.8 ± 9.1	32.3 ± 10.8	0.50
Usual number of days of bleeding	5.9 ± 1.1	6.1 ± 1.1	6.1 ± 1.1	6.5 ± 1.5	6.3 ± 1.1	0.0003
Menstrual cycle phase at time of the study						0.06
Menstrual phase	20 (15.6)	24 (18.8)	24 (18.8)	27 (21.1)	27 (21.1)	
Premenstrual phase	30 (23.4)	34 (26.6)	41 (32.0)	35 (27.3)	39 (30.5)	
Intermenstrual phase	78 (60.9)	70 (54.7)	63 (49.2)	66 (51.6)	62 (48.4)	
Physical activity (total metabolic quivalents-hours/day)	34.3 ± 3.9	33.6 ± 2.1	33.3 ± 1.5	33.9 ± 3.0	34.1 ± 2.8	0.89
Dietary glycemic index	63.6 ± 4.1	65.0 ± 3.7	65.9 ± 4.0	66.3 ± 3.9	66.4 ± 3.6	<0.0001

a Values are means ± SDs or number of subjects (percentages).

b For continuous variables, a linear trend test was used with the median value in each quintile as a continuous variable in linear regression; for categorical variables, a Mantel-Haenszel chi-square test was used.

**Table 3:** MDQ scores in the premenstrual phase according to quintile of dietary hardness in 640 Japanese women aged 18–22 years.^a^

**Variable**	**Quintile 1 *(n* =128)**	**Quintile 2 *(n* =128)**	**Quintile 3 *(n* =128)**	**Quintile 4 *(n* =128)**	**Quintile 5 *(n* =128)**	***P for* trend^b^**
Dietary hardness (mV·s/4184 kJ)	138 (68–148)	160 (149–168)	177 (169–183)	191 (184–198)	211 (199–303)	
MDQ total score (%)^c^	127.3 ± 3.1	124.0 ± 3.0	120.6 ± 3.0	126.5 ± 3.0	129.9 ± 3.0	0.48
MDQ subscale scores (%)^c^						
Pain	132.9 ± 3.8	131.4 ± 3.6	129.1 ± 3.7	132.6 ± 3.7	137.5 ± 3.7	0.40
Concentration	116.1 ± 3.1	114.2 ± 3.0	109.1 ± 3.0	114.5 ± 3.0	116.8 ± 3.0	0.91
Behavioral change	134.3 ± 4.8	125.6 ± 4.6	122.2 ± 4.7	130.8 ± 4.7	134.1 ± 4.7	0.83
Autonomic reactions	107.1 ± 2.4	104.4 ± 2.3	103.5 ± 2.3	109.2 ± 2.3	110.2 ± 2.3	0.18
Water retention	146.5 ± 5.3	145.7 ± 5.1	136.5 ± 5.1	144.7 ± 5.2	144.6 ± 5.1	0.74
Negative affect	130.4 ± 5.0	128.8 ± 4.8	124.2 ± 4.9	131.9± 4.9	136.8 ± 4.9	0.34

a Values are medians (ranges) for dietary hardness and means ± SEs for MDQ scores. MDQ scores in the premenstrual phase were expressed as percentages relative to those in the intermenstrual phase.

b A linear trend test was used with the median value in each quintile as a continuous variable in linear regression.

c Adjusted for age (years, continuous), body mass index (kg/m^2^, continuous), residential block (north: Kanto, Hokkaido, and Tohoku; central: Kinki, Tokai, and Hokuriku; or south: Kyushu and Chugoku), size of residential area (city with a population >1 million, city with a population <1 million, or town and village), current smoking (yes or no), age at menarche (years, continuous), usual length of the menstrual cycle (days, continuous), usual number of days of bleeding (continuous), menstrual cycle phase at the time of the study (menstrual, premenstrual, or intermenstrual), physical activity (total metabolic equivalents-hours/d, continuous), and dietary glycemic index (continuous).

**Abbreviations:** MDQ, Menstrual Distress Questionnaire.
